# FLIRT-ing with Zika: A Web Application to Predict the Movement of Infected Travelers Validated Against the Current Zika Virus Epidemic

**DOI:** 10.1371/currents.outbreaks.711379ace737b7c04c89765342a9a8c9

**Published:** 2016-06-10

**Authors:** Andrew Huff, Toph Allen, Karissa Whiting, Nathan Breit, Brock Arnold

**Affiliations:** Technology & Data Science, EcoHealth Alliance, New York, NY, USA; Technology & Data Science, EcoHealth Alliance, New York, NY, USA; Technology & Data Science, EcoHealth Alliance, New York, NY, USA; Technology & Data Science, EcoHealth Alliance, New York, NY, USA

**Keywords:** air travel, disease forecasting, flirt, networks, Zika, zika virus

## Abstract

Introduction: Beginning in 2015, Zika virus rapidly spread throughout the Americas and has been linked to neurological and autoimmune diseases in adults and babies. Developing accurate tools to anticipate Zika spread is one of the first steps to mitigate further spread of the disease. When combined, air traffic data and network simulations can be used to create tools to predict where infectious disease may spread to and aid in the prevention of infectious diseases. Specific goals were to: 1) predict where travelers infected with the Zika Virus would arrive in the U.S.; and, 2) analyze and validate the open access web application’s (i.e., FLIRT) predictions using data collected after the prediction was made.

Method: FLIRT was built to predict the flow and likely destinations of infected travelers through the air travel network. FLIRT uses a database of flight schedules from over 800 airlines, and can display direct flight traffic and perform passenger simulations between selected airports. FLIRT was used to analyze flights departing from five selected airports in locations where sustained Zika Virus transmission was occurring. FLIRT’s predictions were validated against Zika cases arriving in the U.S. from selected airports during the selected time periods.  Kendall’s τ and Generalized Linear Models were computed for all permutations of FLIRT and case data to test the accuracy of FLIRT’s predictions.

Results: FLIRT was found to be predictive of the final destinations of infected travelers in the U.S. from areas with ongoing transmission of Zika in the Americas from 01 February 2016 - 01 to April 2016, and 11 January 2016 to 11 March 2016 time periods. MIA-FLL, JFK-EWR-LGA, and IAH were top ranked at-risk metro areas, and Florida, Texas and New York were top ranked states at-risk for the future time period analyzed (11 March 2016 - 11 June 2016). For the 11 January 2016 to 11 March 2016 time period, the region-aggregated model indicated 7.24 (95% CI 6.85 – 7.62) imported Zika cases per 100,000 passengers, and the state-aggregated model suggested 11.33 (95% CI 10.80 – 11.90) imported Zika cases per 100,000 passengers.

Discussion: The results from 01 February 2016 to 01 April 2016 and 11 January 2016 to 11 March 2016 time periods support that modeling air travel and passenger movement can be a powerful tool in predicting where infectious diseases will spread next. As FLIRT was shown to significantly predict distribution of Zika Virus cases in the past, there should be heightened biosurveillance and educational campaigns to medical service providers and the general public in these states, especially in the large metropolitan areas.

## Introduction

Zika Virus was first isolated in 1947 in Uganda from a captive rhesus monkey.^1^ Five years later, the first evidence of humans contracting the virus was reported in Uganda and the United Republic of Tanzania.^2^ Over the next 50 years, the virus spread throughout Africa and Asia 3, though only 14 human cases were reported during this time period.^4^ The first major human outbreak of Zika occurred in 2007, in the Federated States of Micronesia on the island of Yap, where approximately three quarters of the 7,391 population contracted the virus.^4^ The most likely source of this outbreak was the inadvertent import of a mosquito vector or via an infected traveler.^4^ Duffy et al. (2009) warned that Zika might easily spread via air travel, citing a medical volunteer who traveled back to the United States after contracting Zika in Yap. The current and ongoing Zika outbreak officially started on May 7, 2015, when Brazil’s National Reference Laboratory confirmed the virus had been isolated in the Americas.^3^ There is active transmission of the Zika Virus in 31 countries in the Americas, with 4,160 confirmed cases and 175,636 suspected cases.^5^^,^ 6

The limited public health and biosurveillance resources available to prepare against Zika must be focused in the locations where the virus is most likely to spread. Developing accurate models to anticipate Zika spread is one of the first steps to mitigate further spread and transmission of the disease. Air traffic data, network models, and simulations have likely improved disease spread forecasting capabilities.^7 ^^,^ 8 ^,^ 9 ^,^ 10 ^,^ 11 ^,^ 12 ^,^ 13 ^,^ 14 Despite the existence of these methods, many of these approaches have not been validated in the peer reviewed literature, but appear to have been useful in predicting the movement of infected travelers over flight networks.

In the 2009 influenza A H1N1 pandemic direct and indirect flight data from the country of origin significantly improved models that predicted when the virus would be detected in other countries. To prevent the further spread of infectious disease outbreaks, software that predicts where infected travelers will travel can be useful to prevent the further spread of infectious diseases. There are publicly available (e.g., GLEAMviz) and non-publicly available (e.g., Center for Disease Control, BLueDoT) software packages and models for flight network analysis; however, validations of these capabilities have not been published in peer review literature. In this manuscript, we validate a new open access web based application against cases of Zika that have arrived in the United States.

## Method

*FLIRT Software*

FLIRT, a biosurveillance application developed under the open source Apache 2.0 license (available at www.apps.eha.io), is designed to predict where infected travelers will likely travel. FLIRT contains a database of flight schedule information that is updated monthly (Innovata 2016) and visualizes passenger flow data over flight networks. Flirt contains two distinct modes: (1) “Scheduled Direct Flights” displays nonstop flight paths from a selected airport to all possible destinations and ranks destinations by summed seat count; and, (2) “Passenger Simulation” displays the results of a Monte Carlo simulation based on passenger layover and transfer probabilities, and was created to mirror real life air travel behavior. All FLIRT results are exportable to JSON, CSV, XML, and XLSX formats.

*Data*

FLIRT uses a database of flights scheduled by over 800 airlines, with each record detailing one scheduled flight route (Innovata, 2016). For each route, the following data are available: (1) operating carrier and call sign; (2) origin and arrival airports; (3) schedule (start and end times, days of week, all repeating weekly); (4) effective and discontinued dates; and, (5) number of seats available. Some scheduled routes operate multiple legs and these routes are split into their constituent flights by interstitial stops, using the relative distance of each leg to impute its departure and arrival time. The data include current, past, and planned routes, extending as far back as October 1, 2014 and continuing into the future as far as 2018. Future scheduled flight routes decreases over time (e.g., approximately half as many routes are scheduled at the start of 2017). At the time of publication, the database contained 54,870,563 flights globally, and tables containing flight counts by region, country, day of the week, and by airport are available in the supplementary materials.

*Nonstop Flight Analysis (FLIRT Direct Scheduled Flights Mode)*

FLIRT assumes that airlines will optimize their planned schedules to meet demand and fill available seats. Thus, FLIRT uses the number of seats available as a proxy for the number of passengers traveling on that route. One degree of edge travel, for a given time period, is calculated by summing all of the seats between two destinations (nodes) for selected time period. An example is provided in Figure 1.

**A screenshot of FLIRT’s interface displaying a network graph based upon scheduled nonstop flights from GRU between 01 February 2016 to 01 April 2016.**
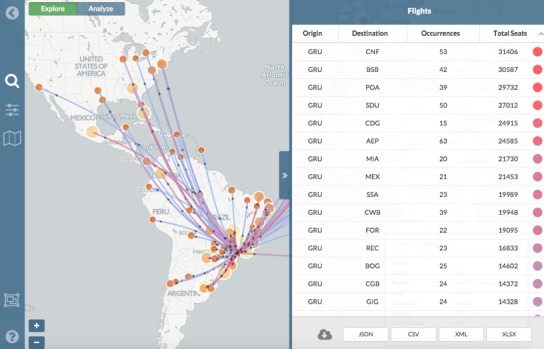

To estimate the global distribution of risk from an outbreak in a location, FLIRT simulates passenger behavior, given these assumptions: (1) infected travelers behave the same as all travelers; (2) the total number of seats scheduled between two locations (nodes) is directly proportional to the number of passengers traveling between those locations (nodes); (3) some travelers take journeys consisting of multiple flights (edges); (4) the probability distribution of the number of edges per trip for all journeys worldwide is the same as that for U.S. domestic flights; (5) travelers on multi edge trips do not double back (i.e., subsequent destinations in a trip that would leave a passenger closer to their origin than to their current location are calculated); and, (6) transfers occur in a temporal window after a passenger arrives at an airport (node) weighted according to a Poisson distribution with λ = 2 hours. If multiple airports are selected, simulated passengers are assigned to each origin airport weighted by their relative total volume of scheduled outgoing seats.

Given a time interval and an origin airport or set of airports, FLIRT simulates trips at random times within that interval, with the above described behavior. Given the previously stated assumption, and the assumption that the disease prevalence of the passengers traveling through the selected airports is equal, the aggregated number of passengers arriving at airports (nodes) should be directly proportional to the rate of arrival of imported disease cases from an outbreak. An example is provided in Figure 2.

**A screenshot of FLIRT’s interface displaying a network graph based upon the simulation of 20,000 passengers departing from CCS between 11 January 2016 to 11 March 2016.**
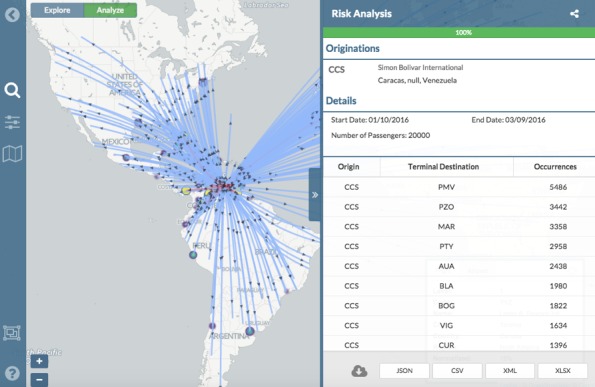

*Interface*

FLIRT users select an airport (node) by searching for name or airport code and selecting from a list of matches. Multiple airports may be selected by: (1) selecting multiple airports from the search interface; (2) automatically including airports in a selectable radius; or, (3) drawing a rectangular selection box on the map. Users also select the start and end date of flight routes, and users select direct scheduled flights (i.e., one-degree connectivity) from the selected airport(s) to all destinations (e.g., Figure 1). Users may also select a passenger simulation after specifying the number of passengers (up to 20,000) they wish to simulate. Results of both modes are displayed with color and thickness scaled routes, as a heat map, and in tabular form. All passenger simulations are cached and may be shared via a unique URL.

*Validation, Verification, Evaluation of FLIRT*

FLIRT’s scheduled direct flights and passenger simulation modes were used to assess records and future schedules of flights departing from five selected origin airports traveling to the continental U.S. over three time periods (Table 1). Origin airports (nodes) were selected based on the number of suspected and confirmed cases per country. As of 02 February 2016, news reports indicated that Brazil, Colombia, El Salvador, Venezuela, and Honduras had the most suspected human cases of Zika Virus. Only international airports were evaluated since only international airports are the only nodes in the network capable of sending infected travelers to the United States from locations with sustained local Zika Virus transmission. The international airport with the most passengers in each Zika-affected country was selected as the origin, as all origin countries had one main international airport at least 2 times the amount of passengers annually as the next busiest international airport in that country (with the exception of Honduras where the busiest airport carried 1.2 times the passenger traffic of the second busiest airport). Regardless of the specific airport that Zika Infected travelers chose to fly, this analysis focused on determining which locations in lower 48 United States are at the highest risk of receiving Zika Infected travelers. The airports selected were Guarulhos International Airport (GRU) in Sao Paulo, Brazil; El Dorado International Airport (BOG) in Bogota, Colombia; Monseñor Óscar Arnulfo Romero International Airport (SAL) in San Salvador, El Salvador; Simón Bolívar International Airport (CCS) in Maiquetia, Venezuela; and Ramón Villeda Morales International Airport (SAP) in San Pedro Sula, Honduras.

**Figure:**
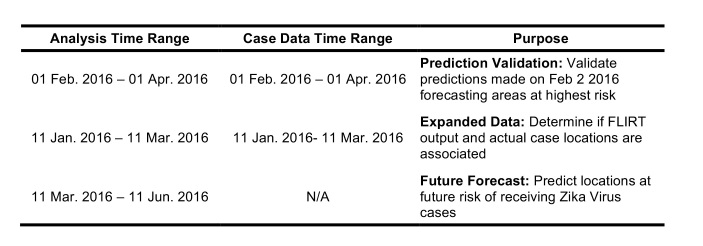
**Table 1**: Three time ranges were chosen to predict and validate Zika case distribution in the continental U.S. The *Prediction Validation* time range was chosen to use up to date case data to validate an earlier future prediction made by the authors using FLIRT data. The analysis using the *Expanded Data* range uses all available case data and matching FLIRT output date ranges for a more in-depth look at FLIRT’s ability to forecast geographic distribution of Zika cases. The *Future Forecast* ranked FLIRT’s results for a future time period to show possible vulnerable areas of future Zika spread in the U.S.

Using FLIRT’s scheduled direct flights mode, individual network maps were generated for each of the five origin airports using counts of seats traveling from selected the origin airports to all possible connected global destinations in each of the three time periods. Then, only the U.S. destination results were extracted and the number of seats from each origin were aggregated to determine the total connectedness between all five origin airports and each possible U.S. destination. Using the passenger simulation, five global simulations were generated for each time range (20,000 passengers per simulation), and each simulation yielded nearly identical results. The results of these five simulations were summed to produce the final simulation results.

To validate FLIRT, FLIRT’s output was compared to the locations of actual U.S. imported Zika cases. Two time ranges were used to assess FLIRT’s ability to predict the rate of imported Zika cases to the U.S during the 2015 Zika Virus epidemic, and one future time range was considered to make future Zika distribution predictions (Table 1). For each time range, direct scheduled flights and passenger simulation results from selected origins to continental U.S. airports, were exported from FLIRT (Table 1). On 02 February 2016, FLIRT predicted a priori which U.S. locations were most at risk of receiving Zika Infected travelers from February 2016 to April 2016, and this prediction was published in The Guardian (Kelkar, 2016). This prediction was validated in this study. The analysis using the Expanded Data range used all available case data collected for this study (163 U.S. Zika cases), and compares it against FLIRT’s 11 January 2016 - 11 March 2016 prediction. The purpose of analyzing the Expanded Data range (a similar time period) was used to further validate FLIRT post hoc, and covered the previous time period merely to avoid the post hoc fallacy.

Case count data were obtained daily and manually from news reports using Google Alerts and searching the Internet from January 11, 2016 to March 11, 2016 using the search terms: (1) new U.S. Zika case; (2) U.S. Zika cases; (3) Zika Virus U.S., and, (4) searches by each state (e.g., Florida Zika Cases). Information about all confirmed and suspected Zika cases and their location was collected; however, detailed geographic information beyond the state level was not always available in the news reports and distinguishing between cases was not difficult due to the heightened media attention surrounding Zika Cases at that time. Most news articles reported heavily on the single first case arriving in a previously Zika-free state. As the Zika Virus epidemic progressed, news articles began reporting on several new cases within a single news article, especially in Zika hotspot states (e.g., Florida, Texas, & New York) where several new cases appeared each day. If specific sub-state level geographic information was available for each case, it was recorded. If not, only state level information was recorded for the cases missing this information. Additional manual internet searches were conducted on cases missing this information to collect missing data and cases were de-duplicated. Real-time reporting alerts from Google Alerts helped the authors identify trends in reporting, like spikes in news activity directly following a new U.S. case finding. Observing these reporting trends helped to identify repeated case reports.

Collected case data was compared to the CDC’s case count information (available at http://www.cdc.gov/zika/geo/united-states.html). Overall, this study’s case data collection matched the CDC’s state level information with two generalizable differences. The CDC had higher case counts for states that contain many cases of Zika (e.g., FL 49 vs. 34). This is partially explained by the longer time frame for which the CDC reported its data (January 1, 2015 – March 9, 2016). Secondly, the data set that was created in this study reported on outbreaks in 6 states the CDC had not reported (AZ, KY, ME, NE, UT, WV). This is most likely because outbreaks occurred in these states after the March 9th cutoff date of the CDC’s available data at the time this study was conducted. Additionally, this data set frequently contained more detailed information (e.g., county level spatial data) which allowed for higher accuracy in associating specific airports within the airport regional analysis.

For comparison with actual case data, and for future predictions of Zika distribution, FLIRT data was exported for the two validation time ranges and grouped by state and airport/metro region. For the state-level analysis, all nonstop flight airport seat counts and simulation results within a state were aggregated at the state level for incoming U.S. flights from Zika affected areas. In the airport/metro regional analyses, each airport code was kept unique, unless the airports were within 60 miles of each other (often representative of large metropolitan regions). Accordingly, JFK – LGA – EWR – HPN, IAD – DCA – BWI, MIA – FLL, and SJC – OAK – SFO were grouped, and all nonstop flights and simulation results for each of these airports were aggregated.

Zika cases were assigned geographic locations based on known location information from news report. Geographic information was available for all Zika cases at least at the state level. Because our simulation algorithms simulate passenger transfer behaviors, which often include transfers to regional domestic flights after international travel, FLIRT’s simulation output includes both regional and international airports as destination results. For analysis of geographic case data against simulation results, we associated each case with whichever airport (regional or international) was closest to the known case location (based on google maps road distance calculated in miles). If only county level information was known, then the largest airport (regional or international) in or most nearby the county center was selected. When geographic information beyond the state level was not known, cases were associated with the highest traffic international state airport. If the nearest airport to a case was within a metro area (airports grouped because they were within 60 miles of each other), the case was associated with the whole metropolitan group (e.g., JFK – LGA – EWR).

Because the selected origin airports are external to the United States, FLIRT’s Scheduled Direct Flights results include international airports almost exclusively. Therefore, for this Zika Virus distribution comparison, the case was associated with the nearest international airport, akin to looking for the case’s likely port of entry. The Zika case was then grouped with the airport to which it was geographically closest. If only state-level geographic information was known about the case, the case was associated with the largest of the two selected state airports. To assess whether the rank order of FLIRT’s predictions corresponded with the rank order of imported Zika caseloads, we computed Kendall’s τ for the same six permutations of data.

*Generalized Linear Models*

This study assumed that the rate of imported Zika cases over time in U.S. locations would be proportional to the number of flights from Zika-affected areas. We tested this with univariate Gaussian general linear models (GLMs) by regressing imported Zika cases against FLIRT’s estimates. We ran these models on all permutations for FLIRT prediction type (one-degree connectedness and multi-degree simulation), time period (restricted to early data and all data), and aggregation level (state and airport region). Before running GLMs, all input variables were standardized by dividing by twice the standard deviation.^15^

To obtain more concretely interpretable coefficients, the 100,000 passenger simulation using actual passenger data for the source airports of interest was rescaled (see supplemental information). These numbers indicate total passengers per year and were divided by two to obtain a rough estimate of outgoing passengers, assuming that: (1) layover passengers are a negligible portion of passengers; and, (2) overestimating the number of outgoing passengers would bias the effect estimates. Multiplying FLIRT’s simulated passenger estimates by the total outgoing passengers over total simulated passengers converts the estimates to passengers per year. The simulations were run on flight data, and matched with cases, for a period of 61 days, this was multiplied by 61 / 365 to obtain a rough estimate of the number of passengers traveling from the selected airports in the time period of observation. We divided this result by 100,000 to obtain a measure of Zika cases per 100,000 passengers from selected airports.

*Future Predictions of Imported Zika Cases*

FLIRT was used to calculate scheduled direct flights (i.e., one degree of edge connectivity) and multi-degree passenger simulations using projected airline schedule data from 11 March 2016 2016 to 06 March 2016. The results of both outputs were compared and states and airport regions were ranked according to their relative air traffic. Global FLIRT results were analyzed to create a rank list to assess the risk of Zika case distribution globally and where U.S. destinations are within these ranks.

## Results

*A Priori Predicted Locations of Imported Zika Virus Cases*

Between 01 February 2016 – 01 April 2016, FLIRT’s explore mode (nonstop flights) identified MIA – FLL, IAH, and ATL – BHM as the airport regions with most passengers arriving from the selected origin airports. At the state level, Florida, Texas, and California were identified as the states with the highest passenger flow from the selected origins. These highest flow metro areas and states were consistently ranked in the same order between 11 January 2016 to 11 March 2016 (Table 3).

FLIRT’s simulation mode (analyze), which more closely mimics real passenger flow, identified MIA – FLL, JFK – LGA – EWR, and IAH as the top destination airports for simulated traffic from the selected origins, and Florida, Texas, and New York as the destination states with the most simulated traffic for the 01 February 2016 to 01 April 2016 (Table 2). The metro region simulation and state results were consistent (11 January 2016 to to 11 March 2016, Table 4).

**Figure:**
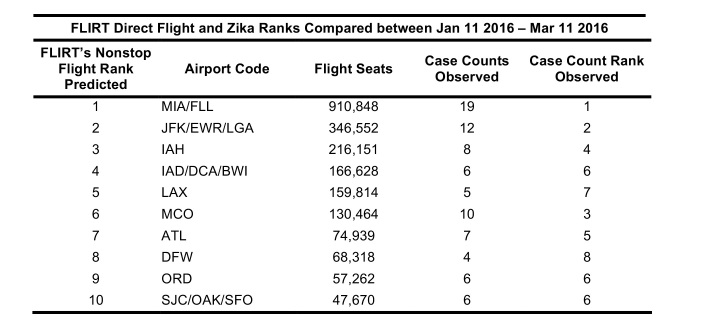
**Table 2**: The top 10 ranked airports with the highest direct flight volume compared to states ranked according to number of Zika virus cases observed from 11 Jan. 2016 – 11 Mar. 2016 and Kendall’s т for the various permutations of aggregation and FLIRT output type are displayed. All rankings are significantly correlated (p<0.05).

**Figure:**
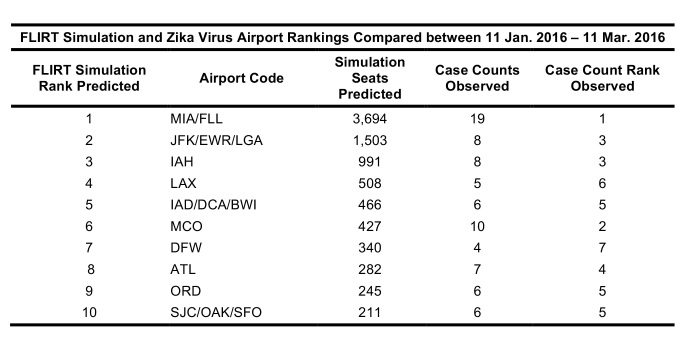
Table 3: Top 10 ranked airports with most simulated passengers compared to states ranked according to number of Zika cases per state for date range 11 Jan. 2016 – 11 Mar. 2016.

**Figure:**
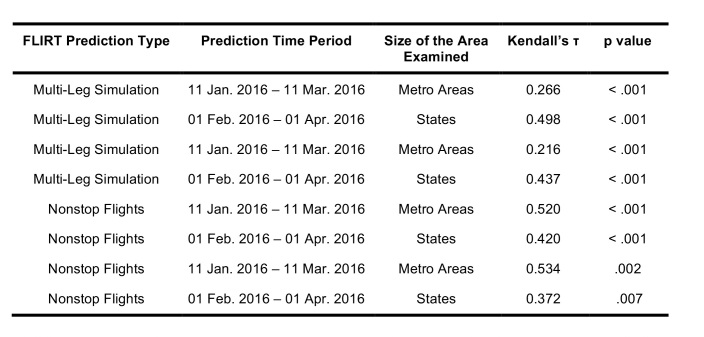
**Table 4**: A comparison of the ordinal ranks of locations receiving imported cases of Zika virus predicted by FLIRT, the observed time period, and the size of the region analyzed. FLIRT’s nonstop flight and multi-leg simulation were both predictive of where Zika cases were most likely to arrive at the state and metro area levels for the observed time periods.

The generalized linear model output also shows a significant association between the number of flights from Zika-affected areas and Zika cases. Between 11 January 2016 – 11 March 2016, the region-aggregated model indicated 7.24 (95% CI 6.85 – 7.62) imported Zika cases per 100,000 passengers, and the state-aggregated model suggested 11.33 (95% CI 10.80 – 11.90) imported Zika cases per 100,000 passengers (Table 5).

**Figure:**
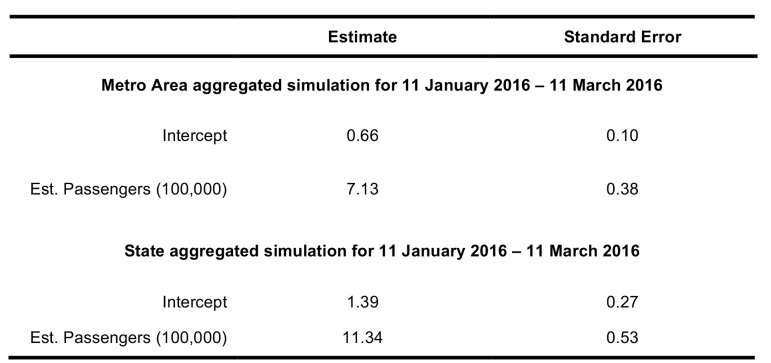
**Table 5**: The intercept and standard error for general linear models for state and metro area aggregated models. A comprehensive listing of linear models, including models of previous estimates with restricted date ranges, and models for nonstop flight data, using standardized confidence intervals, are available in Supporting Information.

*Future Prediction of Imported Zika Cases*

The future FLIRT evaluation (Table 6 and 7) identified MIA – FLL, JFK – EWR – LGA, and IAH as having both highest traffic flight levels and top simulation results from the selected origins. Consequently, Florida, Texas and New York had the highest direct flight traffic and simulation values. The simulation rankings for the Future Forecast time period were consistent with the earlier time period ranks. While consistent with the Future Forecast simulation results, the Future Prediction results differed slightly from previous direct flight time period rankings where MIA – FLL, IAH and ATL – BHM were the top identified metro regions, and Florida, Texas and California were top state. Aggregation of all airports within a state does not change the rank order to a large degree (Table 6 and 7).

**Figure:**
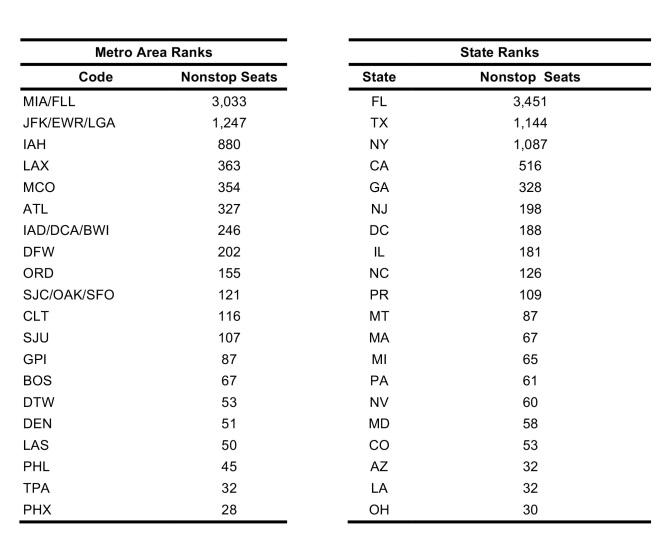
**Table 6**: The top 20 results from a simulation of 100,000 passengers from five origin airports between 11 March 2016 and 11 June 2016. Aggregation of all airports within a state does not change the rank order to a large degree.

**Figure:**
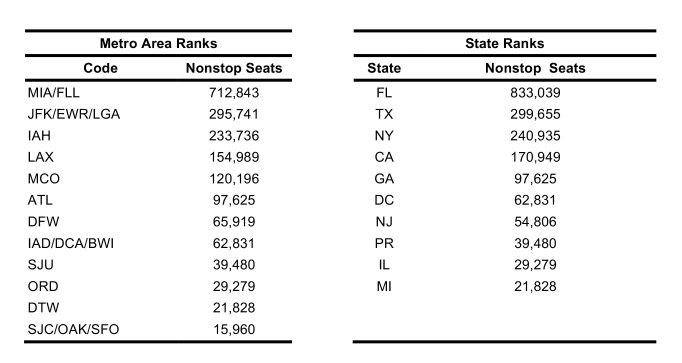
**Table 7**: Nonstop flights ranked from five origin airports for 3.11.2016 – 6.11.2016 time period. The top 20 results from scheduled nonstop flights from five origin airports between 11 March 2016 and 11 June 2016. Aggregation of all airports within a state does not change the rank order to a large degree.

Global airports at risk were also ranked between 11 March 2016 – 11 June 2016. According to the simulations, Tocumen International Airport in Panama City, Panama, had the highest probable incoming passengers from the five origin airports (GRU, BOG, SAP, SAL, CCS). U.S. airports were not on the top 10 ranked simulation list (Table 8). However, in the direct flight global ranking Miami International Airport and George Bush Intercontinental Airport ranked in the top ten airport destinations (Table 9). Tocumen International Airport, Panama; Rafael Nunez International Airport, Colombia; Alfonso Bonilla Aragon International Airport, Colombia; and Santiago Marino Caribbean International Airport, Venezuela all ranked on both direct flight and simulation top 10 lists.

**Figure:**
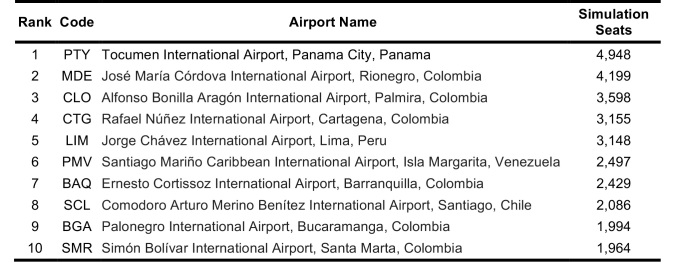
**Table 8**: The top 10 global destinations from chosen origins with sustained Zika virus transmission based on a simulation of 100,000 passengers between 11 March 2016 and 11 June 2016.

**Figure:**
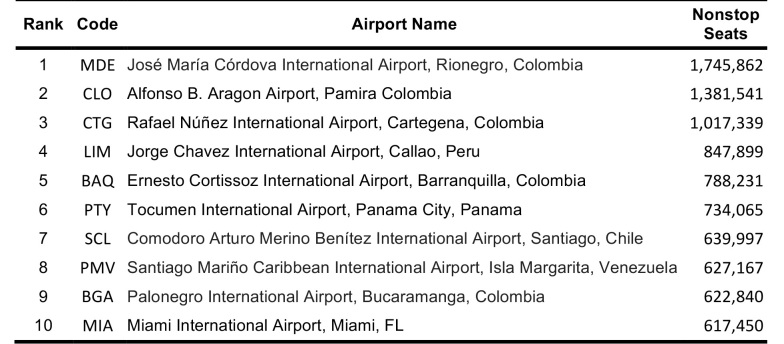
**Table 9**: The top 10 global destinations from chosen origins with sustained local Zika virus transmission in the Western hemisphere based on the scheduled nonstop flights between 11 March 2016 and 11 June 2016.

## Discussion

The emergence of Zika Virus in the Americas is a reasonable way to test FLIRT’s predictive ability since it is most likely that many Zika Virus cases are imported via air travel. Although there is now mounting evidence of sexual transmission of Zika Virus, the ecological niche of vectors (i.e., A. aegypti and A. albopictus) only covers southern portions of the continental U.S. and sustained transmission has not yet been reported in this area, thus these other types of transmission are not likely to bias this study’s results.

In early February of 2016, FLIRT was used to predict the arrival of travelers infected with Zika Virus in the U.S. traveling from locations in the Americas with sustained transmission from 01 February 2016 to 01 April 2016. FLIRT predicted a priori that Florida, Texas, New York, and California (respectively) would be at the highest risk of receiving travels infected with Zika Virus, with Miami, Houston, New York, and Los Angeles metropolitan areas most at risk. News reports of Zika case occurrences in the U.S. since that time have confirmed FLIRT’s predictions; from 01 February 2016 to 01 April 2016 that Florida, Texas, and California would receive the most cases of Zika Virus from the Americas. The Kendall T statistic between ranked case and simulation lists for this time range was significant in all conditions and the generalized linear models supported the hypothesis that FLIRT is predictive of locations where Zika infected travelers would arrive in the U.S. from 01 February 2016 to 01 April 2016.

To expand this study’s analysis, and better determine the accuracy of future predictions, this study also collected all available Zika case data available from 11 January 2016 to 11 March 2016 and repeated the analysis. The generalized linear model results for this time range produced smaller standard errors than between 01 February 2016 and 01 April 2016. This result indicated that when more data were included, the fit of the model improved. The results from 01 February 2016 to 01 April 2016 and 11 January 2016 to 11 March 2016 support that modeling air travel and passenger movement can be a powerful tool in predicting where infectious diseases will spread next.

Due to FLIRT’s predictive ability, this study predicts areas that will be at higher risk of receiving travelers infected with the Zika Virus in the U.S. (Tables 6, 7, & 8). FLIRT’s direct scheduled flight analysis and passenger simulation results predict that (10 March 2016 to 01 June 2016) MIA – FLL, JFK – EWR – LGA, and IAH will continue to be at highest risk of receiving Zika infected travelers, and Florida, Texas, and New York will be the states most at risk. As FLIRT was shown to significantly predict distribution of Zika Virus cases in the past, there should be heightened biosurveillance and educational campaigns to medical service providers and the general public in these states, especially in the large metropolitan areas. Despite these areas being at the most risk of receiving travelers infected with Zika Virus, the environmental conditions at these locations may not be conducive to sustained local transmission 16

Case reports of Zika were commonly reported at the state, county, or metro area level, and specific travel information (like where infected travelers arrived in the U.S.) was difficult to ascertain. This forced the assumption that infected travelers that arrived in the U.S. used airports within close proximity to where they resided or where they were diagnosed with the disease. It is possible that people diagnosed with Zika Virus used other modes of transportation, or multiple modes of transportation, before arriving at the location where they were diagnosed with disease. Better infected traveler information about how Zika cases arrived in the U.S. could ameliorate these concerns. Often, precise case locations were not publically available, and the CDC only released state level information about U.S. cases. The rate of imported cases to specific airports in the U.S., from the origin airports analyzed in this study, is partially dependent on the prevalence of Zika Virus in the source population. If Zika prevalence varied greatly between different airports, such that their number of cases per flight was vastly different, it could alter the distribution of cases in the United States. However, based upon the origin airports and the time periods analyzed in this study, it is unlikely that large differences in prevalence would overwhelm the effects of the network’s structure in this validation of FLIRT since many of the origin airports have similar network structure and passenger volume (see supplementary materials).

In the future, this study should be expanded globally and FLIRT should be further validated against other future instances of infectious disease emergence. Similarly, FLIRT should be validated against other past outbreaks (e.g., Ebola Virus, SARS, MERS) to determine how predictive it is under other conditions. With further validation against other known infectious disease events, FLIRT will be a powerful tool to aid policy-makers and inform resource allocation to prevent and mitigate disease risk worldwide. As Zika Virus expands across suitable environments, understanding travel networks is increasingly critical in the highly connected and fast moving world.

## Data Availability Statement

Our flight data is publicly available to all researchers (i.e., flirt.eha.io). Zika Virus case data is available in the manuscript in tables.

## Competing Interests

We have read the journal's policy and have this potential conflict: This study was made possible by the generous support of Defense Threat Reduction Agency (DTRA) through a contract (Contract No. HDTRA1-13-C-0029) awarded to Dr. Andrew Huff while employed at EcoHealth Alliance. The contents are the responsibility of the authors and do not necessarily reflect the views of EcoHealth Alliance, DTRA, or the United States Government.

## Supporting Information

**Figure:**
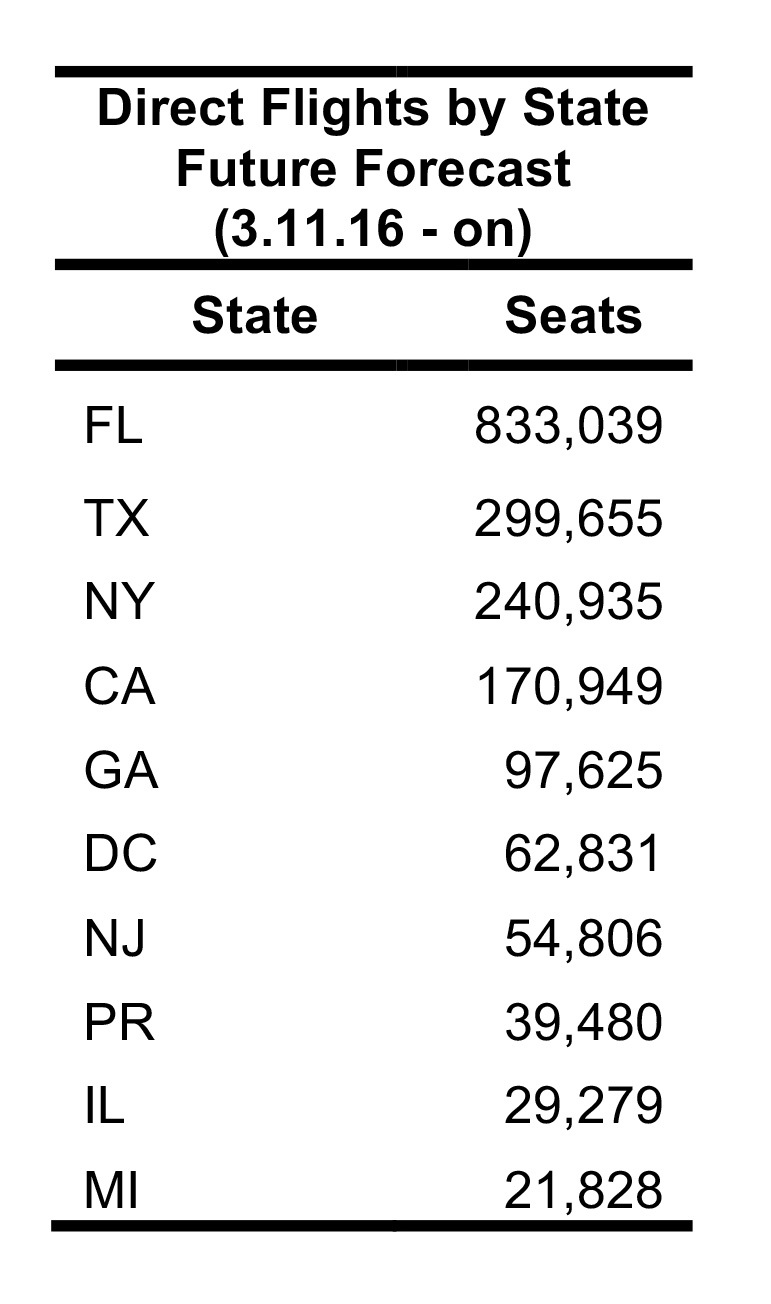
S1 Table

**Figure:**
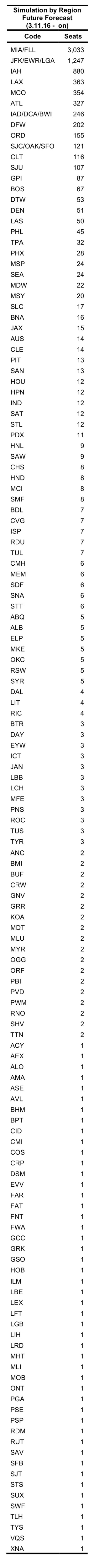
S2 Table

**Figure:**
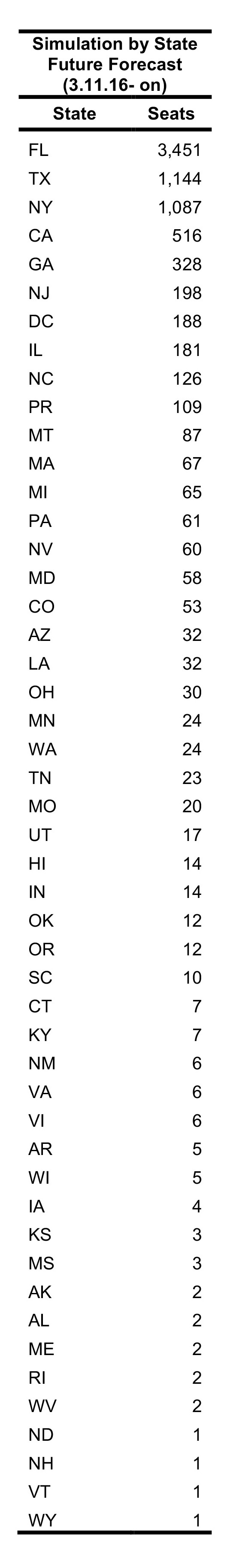
S3 Table

**Figure:**
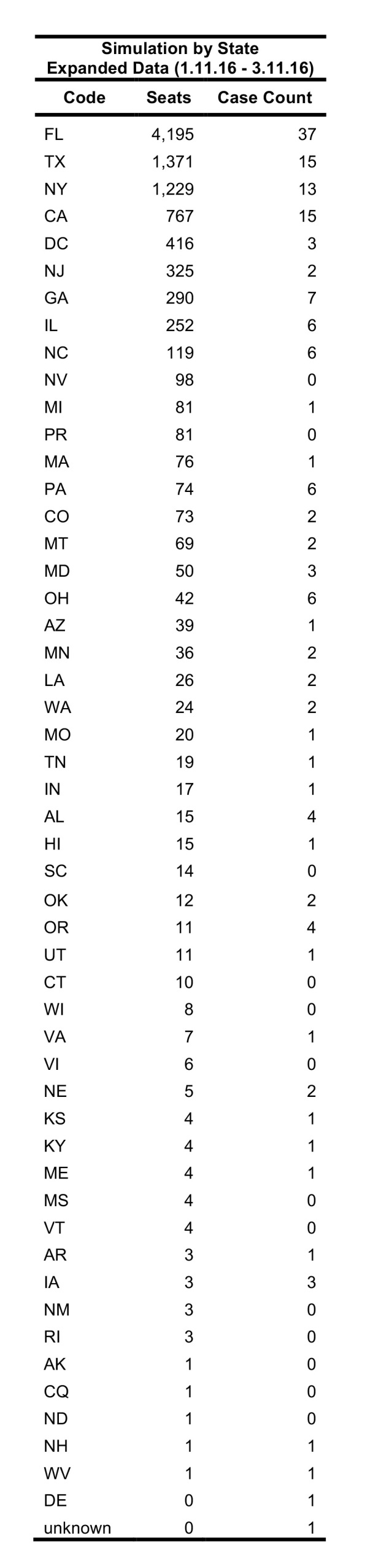
S4 Table

**Figure:**
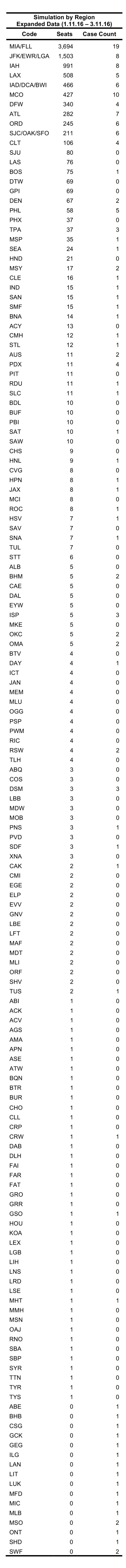
S5 Table

**Figure:**
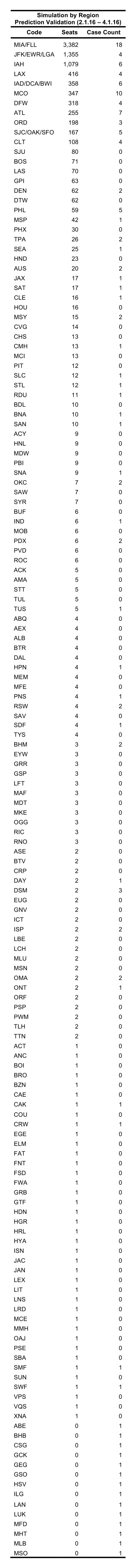
S6 Table

**Figure:**
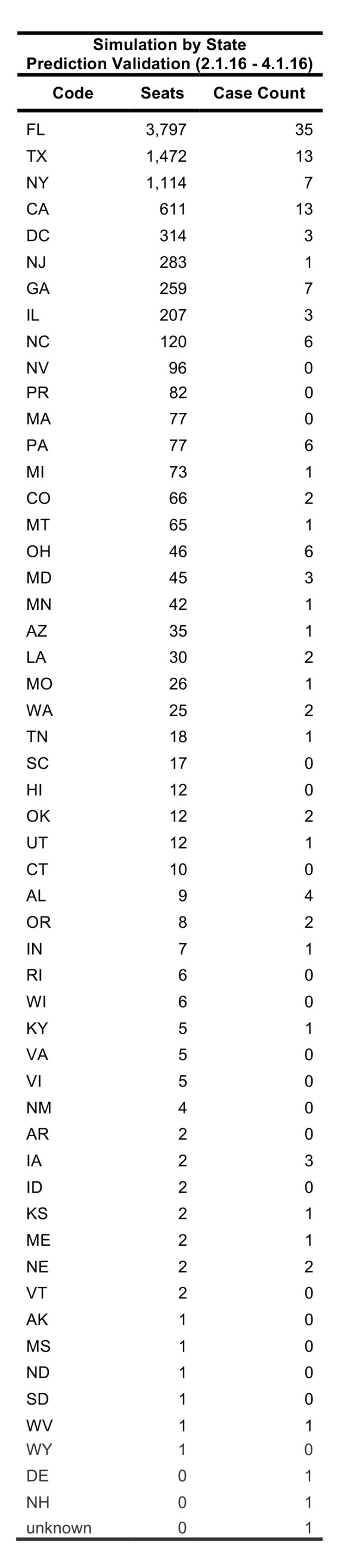
S7 Table

**Figure:**
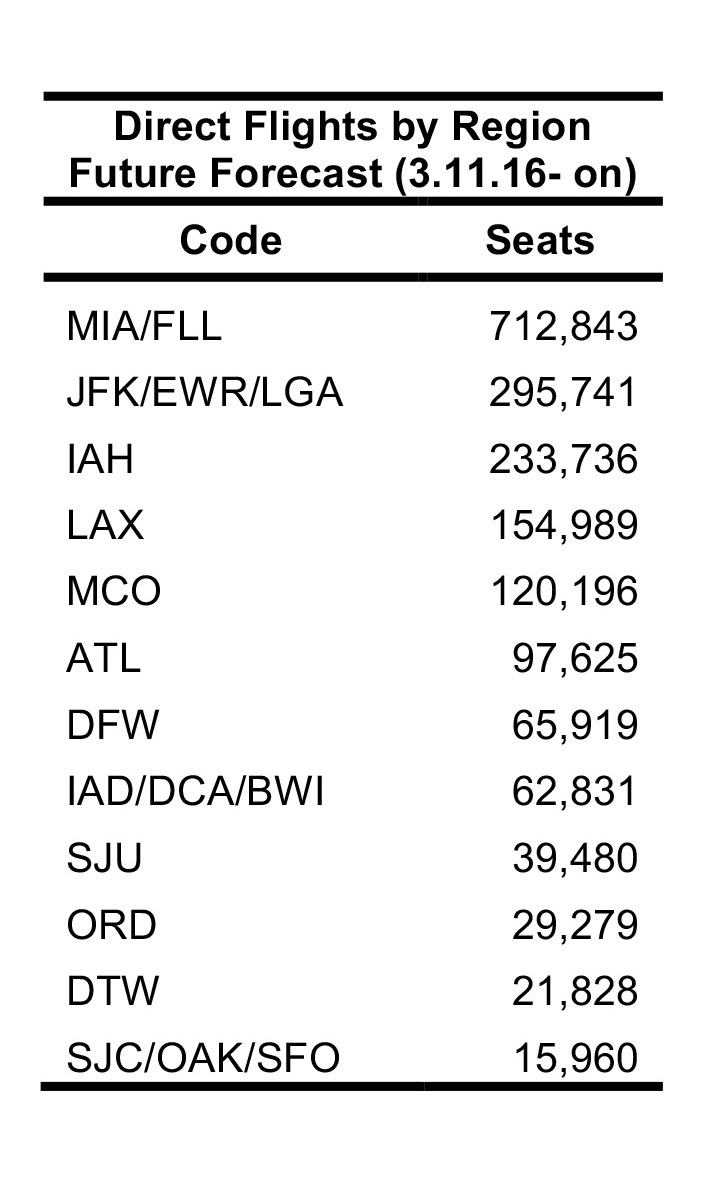
S8 Table

**Figure:**
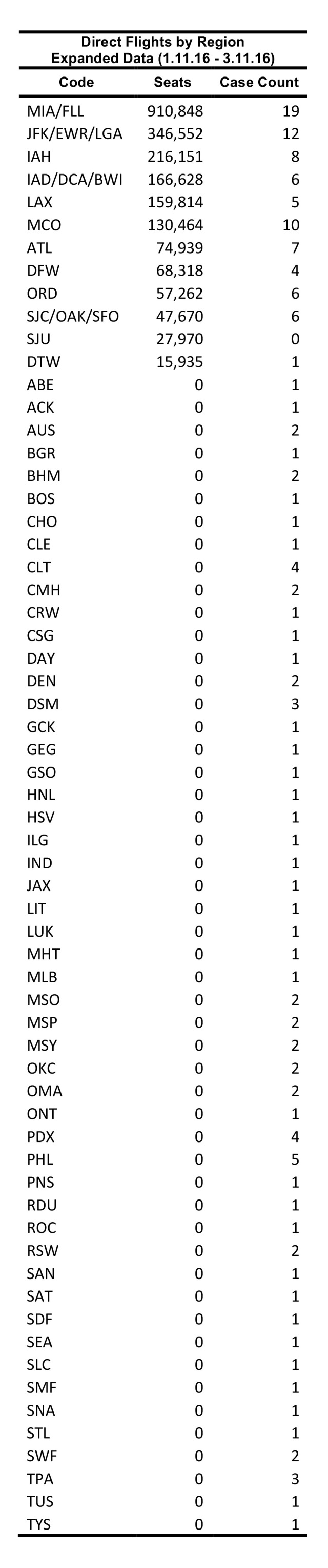
S9 Table

**Figure:**
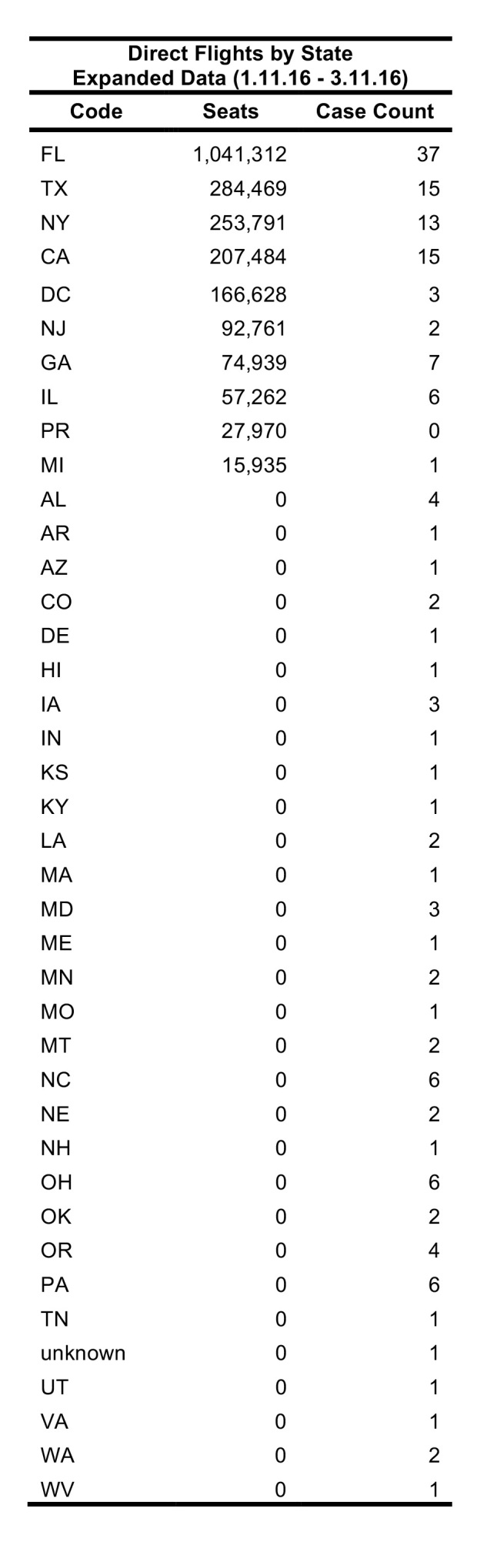
S10 Table

**Figure:**
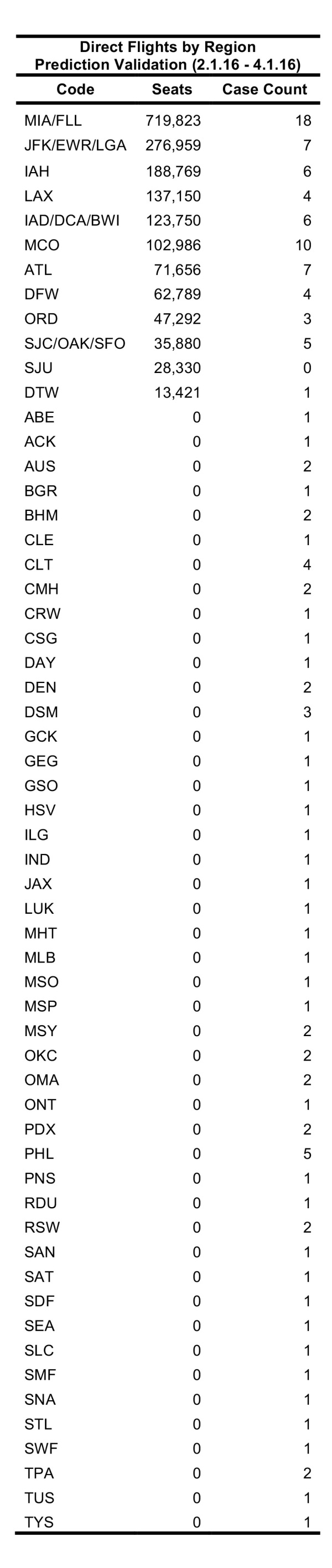
S11 Table

**Figure:**
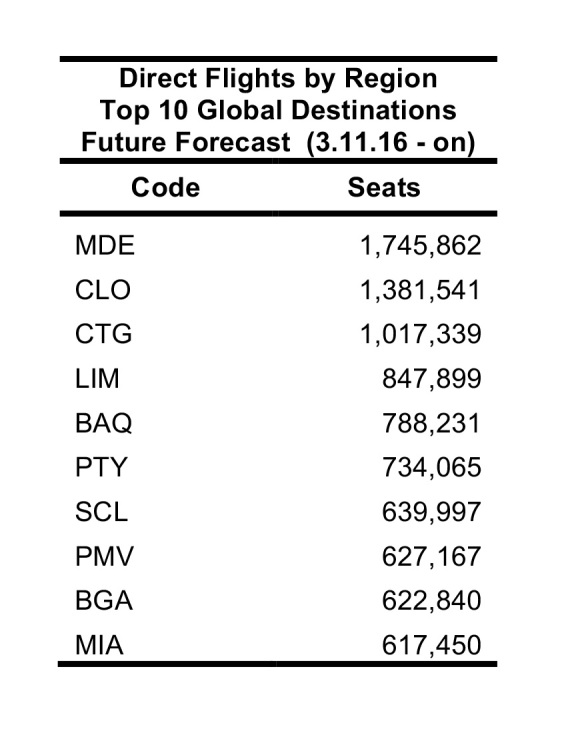
S12 Table

**Figure:**
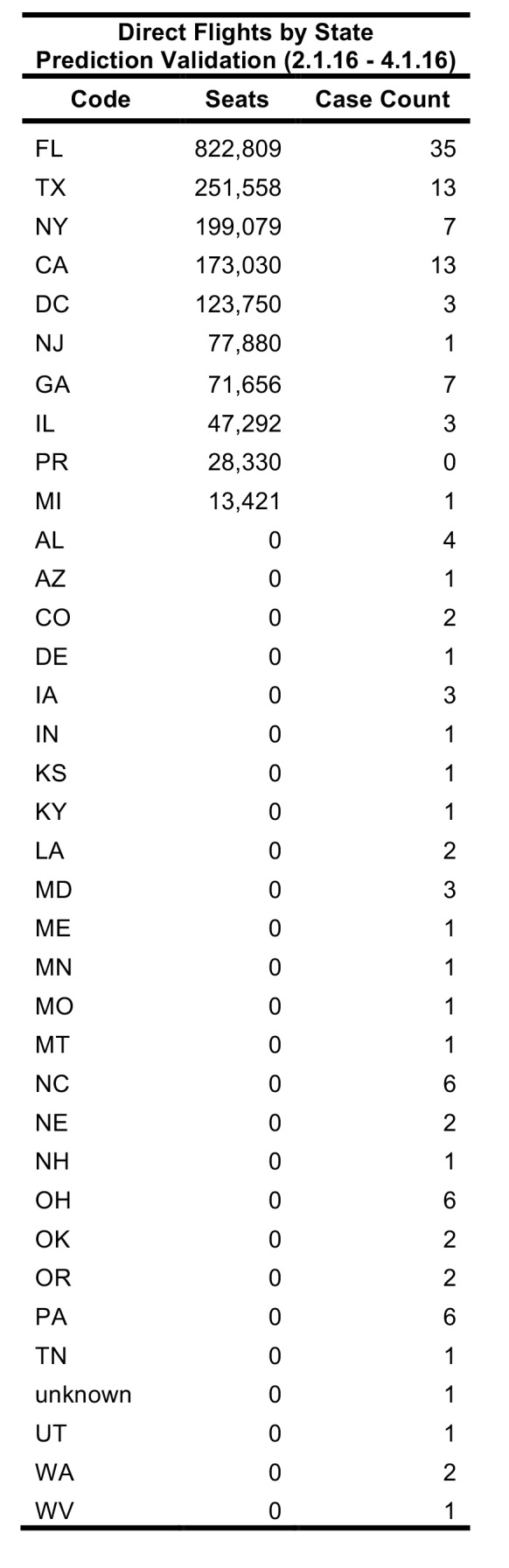
S13 Table
